# Harnessing catalytic pumps for directional delivery of microparticles in microchambers

**DOI:** 10.1038/ncomms14384

**Published:** 2017-02-17

**Authors:** Sambeeta Das, Oleg E. Shklyaev, Alicia Altemose, Henry Shum, Isamar Ortiz-Rivera, Lyanne Valdez, Thomas E. Mallouk, Anna C. Balazs, Ayusman Sen

**Affiliations:** 1 Department of Chemistry, The Pennsylvania State University, University Park, Pennsylvania 16802, USA; 2 Department of Chemical Engineering, University of Pittsburgh, Pittsburgh, Pennsylvania 15261, USA

## Abstract

The directed transport of microparticles in microfluidic devices is vital for efficient bioassays and fabrication of complex microstructures. There remains, however, a need for methods to propel and steer microscopic cargo that do not require modifying these particles. Using theory and experiments, we show that catalytic surface reactions can be used to deliver microparticle cargo to specified regions in microchambers. Here reagents diffuse from a gel reservoir and react with the catalyst-coated surface. Fluid density gradients due to the spatially varying reagent concentration induce a convective flow, which carries the suspended particles until the reagents are consumed. Consequently, the cargo is deposited around a specific position on the surface. The velocity and final peak location of the cargo can be tuned independently. By increasing the local particle concentration, highly sensitive assays can be performed efficiently and rapidly. Moreover, the process can be repeated by introducing fresh reagent into the microchamber.

## Abstract (web summary)

Targeted delivery of microparticles is desirable for rapid, sensitive biological assays or self-assembly process. Here Das *et al*. use catalytic reactions on the surface of microfluidic chambers to generate unidirectional flows that carry and deposit microparticles to selective regions of the chamber.

The targeted transport and delivery of microparticles (for example, cells, bacterial spores and colloids) to specific locations in fluid-filled microchambers is essential for applications ranging from biological assays to bottom-up assembly of complex structures^1^,^2^,^3^,^4^. Directed transport of analytes is especially vital for performing rapid, sensitive assays since diffusion alone cannot provide timely accumulation of these species at localized sensors^5^. This has been demonstrated for a model system by Sheehan and coworkers: relying purely on diffusive transport, femtomolar concentrations of 20-base single-stranded DNA molecules require a timescale of minutes or longer to encounter a typical microscale sensor^5^. Detecting much larger, micron-scale particles (for example, cells, spores and viruses) by diffusion alone would be orders of magnitude slower and, therefore, impractical.

Active transport, where chemicals in the environment can guide particle motion, can alleviate this problem because higher signal/noise ratios can be achieved in a shorter time. Recently, progress has been made in harnessing chemical gradients in fluids to control the movement of molecules and nanoparticles, achieving millimetre-scale transport over several hours^6^,^7^,^8^. As the size of the particles increases, however, the performance of techniques based on molecular gradients progressively decreases, and hence, there is a vital need for better methods to steer microparticles in solution, for example, by microfluidic flows. In microfluidic systems, actuation forces such as external pressure and thermocapillary effects are typically used to create flows that propel particles up to 100 body lengths per second^9^; such external inputs could, however, damage sensitive biological analytes. Electric fields and magnetic fields can also be used to transport and focus analytes, though charged particles or magnetically tagged analytes are required for this application^10^,^11^. Recently, the diffusioosmotic effect was used to deliver micron size particles into dead-end microchannels, where conventional pressure-driven mechanisms are inapplicable^12^,^13^. Diffusioosmotic fluid flow is controlled by properties of the channel surface and the magnitude of the chemical gradients. Hence, the mechanism is effective at small length scales but is difficult to scale-up and control. Other methods utilize motor proteins, such as kinesins, which can transport protein-bound analytes^3^. The binding of analytes to motor proteins also requires specialized particles or surface modification of desired analytes. While self-propelled microparticles can be fabricated^14^, again, a more general approach is needed that does not involve modifying the particles to be analysed.

Herein, we propose a method for microparticle transport that represents a unique advantage over the previously mentioned strategies since convective flow transport does not damage or require modification of the analytes. We use both theory and experiment to demonstrate that simple chemical reactions will induce unidirectional fluid flows that carry micron-sized particles towards a specific region in space. Through these studies, we show that chemically induced fluid pumping enables the repeatable delivery of microparticles to targeted surface sites in microchambers.

## Results

### Reaction driven microfluidic pumps

In designing our system, we take advantage of the recent finding^15^ that enzyme molecules immobilized on a surface within a fluid can convert chemical energy released by a reaction with dissolved reagents into mechanical motion of the surrounding fluid, and thereby, act as a pump, which generates microscale convective flows. The pumping velocity can be increased by increasing the concentration of reagents in the solution and the reaction rate^15^. A variety of such microscale pumps have recently been developed^15^,^16^,^17^,^18^,^19^,^20^,^21^,^22^,^23^,^24^,^25^ by anchoring catalysts to surfaces in solution. To date, however, these micropumps have primarily been utilized to generate radial, re-circulating flows, which are not suitable for transporting and localizing particles at specified distances away from the pump. Here we introduce a source of the reagent at one end of the microchamber and investigate the system's ability to deliver microparticles to a targeted site on the surface. In particular, we introduce a gel that is soaked with reagent at a distance from the catalyst-decorated region in the chamber. The reagents diffuse out of the gel and into the surrounding fluid, producing a concentration gradient across the underlying catalytic surface. As we show below, this concentration gradient leads to asymmetric flow patterns that transport cargo and deposit this cargo at specific locations within the chamber.

The above approach provides a number of advantages. In particular, sensors typically require a critical threshold concentration to effectively detect a given compound; our approach enables a relatively large amount of an analyte to be autonomously delivered and concentrated at a sensor at a particular location on the surface, allowing the sensor to readily recognize these species^5^. The fluid velocity and the rate of cargo transport can be controlled by tuning the concentration of reagent in the gel (or the porosity of the gel), as well as the rate of reaction. After all the reagent has been consumed, the pumping stops and particles sediment to the desired location. Importantly, the fluid motion can be restarted by adding fresh reagent into the gel and hence, the system can be used repeatedly.

Below, we describe our model for these catalytic micropumps, discussing the physical mechanism underlying the convective transport of particles. We then detail the results of numerical simulations and the corresponding experiments that corroborate our findings. On a conceptual level, these findings reveal how fluid flow (and the suspended particulates) in a confined chamber can be patterned and manipulated on-demand by varying readily accessible parameters in the system.

### Theoretical model for the convective transport of particles

The mechanism of transducing chemical energy into fluid motion demonstrated by Sengupta *et al*.^15^ can be utilized for on-demand transportation of micron-sized cargo suspended in the fluid. This mechanism is based on the chemical decomposition of a reagent into products by surface-bound catalysts, which generates a local density variation, which in turn gives rise to fluid flows. The density variation of an aqueous solution, comprised of solvent of density *ρ*_0_ with *N*^C^ chemical solutes characterized by corresponding concentrations *C*^*j*^, can be approximated as


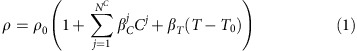


This expression also accounts for possible variations due to a temperature change (*T*−*T*_0_; measured with respect to reference value *T*_0_), resulting from endothermic or exothermic chemical reactions. The expansion coefficients 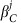 and *β*_*T*_ characterize the magnitude of density variations in response to changes in solute concentrations *C*^*j*^ and temperature (*T*−*T*_0_).

**Figure 1:**
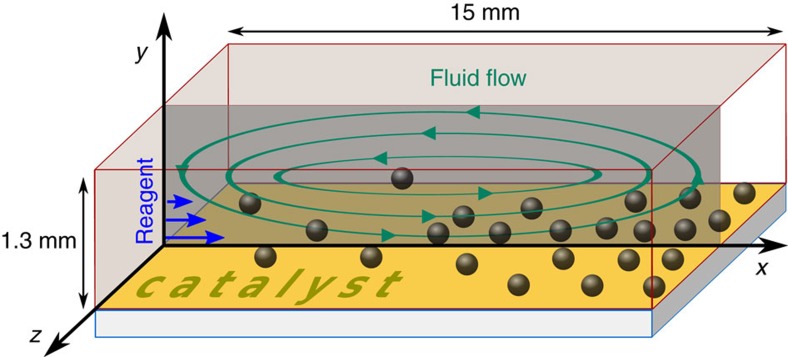
Geometry of the simulation domain. Reagent (released out of gel) enters the domain through the left wall at *x*=0. The catalyst, coating the region on the bottom, decomposes the reagent into lighter products. The resulting density variation across the domain generates convective flows, transporting the tracers.

To demonstrate the physical mechanisms of convective transport, we choose a specific realizable system. In particular, we consider convection in the long, narrow channel schematically shown in Fig. 1 and, as a representative example of a catalytic chemical reaction, we choose the well-studied decomposition of an aqueous solution of hydrogen peroxide by a catalyst (for example, platinum or catalase) to form oxygen and water: 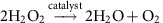. Here catalase was chosen as the model catalyst, since the essential reaction parameters are known. At low hydrogen peroxide concentrations, 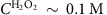, the chemical reaction is accompanied by the production of small concentrations of oxygen, 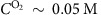, and a temperature increase of (*T*−*T*_0_)∼0.1–1 K. Combining these values with the solutal expansion coefficients for hydrogen peroxide, 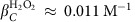, and for oxygen, 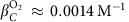, as well as the thermal expansion coefficient for water, *β*_*T*_∼10^−4^ K^−1^, we find that the solution density variation due to hydrogen peroxide concentration, 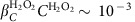, is approximately an order of magnitude larger than the contribution due to the oxygen concentration, 
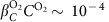
, and thermal effect *β*_*T*_(*T*−*T*_0_)∼10^−4^. Notably, the products of the reaction are lighter than the reagents. We, therefore, simplify the density expression in equation (1) to 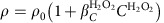. An analogous simplification can be applied to other relevant catalytic chemical reactions^15^ by using the appropriate reaction kinetics parameters and expansion coefficients.

Cargo immersed in the fluid is modelled as spherical polystyrene tracers of radius *R*=2 μm and density *ρ*_t_=1.05 g cm^−3^. Assuming the regime of slow fluid velocities, in which particles do not alter the fluid flow or interact with each other, the motion of each tracer is determined by the superposition of advection with the local fluid flow **u**, gravitational sedimentation with speed 
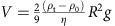
 and Brownian motion, characterized by the tracer diffusion coefficient 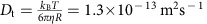. Here *g* is the acceleration due to gravity, *η* is the dynamic viscosity of the host fluid, *k*_B_ is the Boltzmann constant and *T* is the absolute temperature. Tracers are prevented from escaping the computational domain by including repulsive interactions with the walls as discussed below. We ignore friction between the individual tracers, and between the tracers and the wall.

The system is symmetric about the *x*–*y* plane (see Supplementary Figs 1 and 2, and Supplementary Movies 1 and 2 for results from three-dimensional simulations) and, thus, we perform simulations in a two-dimensional rectangular domain {(*x*, *y*): 0≤*x*≤*L*, 0≤*y*≤*H*}, which has length *L* and height *H*. The dimensions of the domain (Fig. 1) match the experimental set-up described further below. The domain is filled with water of density *ρ*_0_=10^3^ kg m^−3^ and kinematic viscosity *ν*=10^−6^ m^2^ s^−1^. We assume that a gel soaked with hydrogen peroxide is located to the left of the computational domain shown in Fig. 1. The hydrogen peroxide enters through the left wall and diffuses throughout the domain with a diffusivity of *D*=10^−9^ m^2^ s^−1^. The fluid motion is described by velocity **u**=(*u*_*x*_, *u*_*y*_) and pressure *p*, the reagent concentration field is given as *C*, and position of *N* tracers is specified by vectors **r**_*i*_(*t*)=(*x*_*i*_(*t*), *y*_*i*_(*t*)). The respective governing equations, equations (2, 3, 4, 5), are the continuity, Navier–Stokes (in the Boussinesq approximation), reagent diffusion and Langevin equation in the overdamped limit:


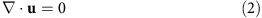



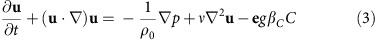



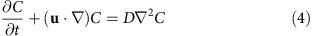



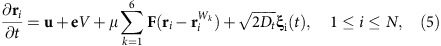


where ∇ is the spatial gradient operator and **e**=(0,1) is a vector specifying the upward vertical direction. Stochastic fluctuations 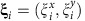 are assumed to be Gaussian white noise,


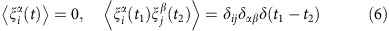


with 1≤*i*, *j*≤*N*, *α*, *β*=*x*, *y*. In equation (5), *μ*=(6*πηR*)^−1^ is the tracer mobility, 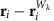 specifies the vector from the tracer to the closest point on the wall labelled *k*, and 
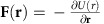
 is the repulsive force between tracers and walls, modelled via Morse potentials


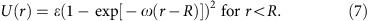


Parameters characterizing the potential strength and width were set to *ɛ*=1.2 × 10^−14^ J and *ω*=6 × 10^4^ m^−1^, respectively.

Decomposition of an aqueous solution of hydrogen peroxide by catalase into oxygen and water occurs over a region of the bottom surface, *y*=0, and is modelled via the Michaelis–Menten relation with the reaction rate


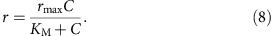


Here the maximal reaction rate *r*_max_=*Mk*_cat_[*E*] incorporates the number *M*=4 of active sites per enzyme molecule, the reaction rate per active site^26^ *k*_cat_=2.12 × 10^5^ s^−1^, and areal enzyme concentration [*E*]=2 × 10^−8^ mol m^−2^ (with the radius of a catalase molecule assumed to be *R*_cat_=5.1 × 10^−9^ m) in moles per unit area. In addition^26^, *K*_M_=0.093 M is the reagent concentration at which the reaction rate is half of *r*_max_.

Using equation (8) and the diffusion flux **j**=−*D*∇*C*, the reagent decomposition in region *A* can be described by 
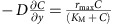
. There are various ways to introduce reagents into the microchamber. In our experiments, we allowed a finite amount of reagent to effuse from an agar gel soaked with the reagent (as described in the experimental section). To separate the physics of the reagent injection into the domain from the mechanism of convective transport, we considered the influx of the reagent into the computational domain through the left boundary of the microchannel. Three boundary conditions were tested for this boundary: (A) exponentially decreasing reagent concentration *C*∼exp(−*t*); (B) linearly decreasing concentration *C*∼1−*t*; (C) exponentially decreasing flux of the reagent into the channel 
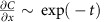
. All of these forms decrease with time, reflecting the depletion of the reagent supply. These different boundary conditions produced qualitatively similar results, indicating that the essential physics of the transportation process is not sensitive to the precise time evolution of the reagent influx rate. We chose to present results for the first type of boundary condition A and specified the reagent concentration to be decaying with time as *C*(*x*=0, *t*)=*C*_0_exp(−*λt*) along the left wall *x*=0. To match experimental observations, the characteristic time of fuel depletion from the gel was set *λ*^−1^=70 min and the initial reagent concentration in the gel was chosen *C*_0_=0.1 M. Other walls are assumed to be impermeable to the reagent. The requirement of zero velocity at the walls, combined with the conditions on the concentration field, yields the full set of boundary conditions:


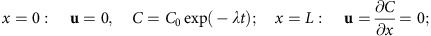



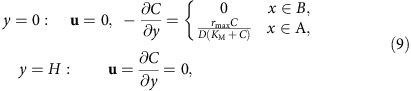


where *A* is the catalyst-covered region of the bottom surface and *B* is the uncovered part.

In our experiments (discussed further below), we used a pattern of functionalized stripes instead of a solid patch. The stripes allowed the tracer particle motion and accumulation to be viewed using an inverted microscope, whereas a solid patch of catalyst would have obstructed the view. Control simulations with discrete stripes of catalyst revealed that when the distance between the stripes is much smaller than the height of the chamber, as was the case in our experiments, the resulting flow field is the same as when the catalyst uniformly covers the surface (Supplementary Note 1). Namely, these closely spaced stripes do not lead to noticeable vortices in the gaps between stripes and the stripe orientation does not affect the flow details.

Equations (2, 3, 4, 5), along with the corresponding boundary conditions, equation (9), were solved as follows. First, a lattice Boltzmann algorithm^27^ was applied to simulate the continuity (equation (2)) and Navier–Stokes (equation (3)) equations. Then, a second-order finite difference scheme was used to solve the diffusion terms in equation (4). At each time step, the computed flow field **u** is used for the chemical advection term in equation (4). The updated concentration field is then used for the buoyancy forces in equation (3) to advance the lattice Boltzmann scheme to the next time step. The computed flow field **u** is also used to solve the equation for tracer motion, equation (5), with a first-order integration scheme. Initially, *N*=500 tracers are randomly distributed throughout the domain and the initial reagent concentration is set to *C*(*t*=0)=0. The findings from these simulations are described below.

### Physical mechanism of convective particle transport

**Figure 2:**
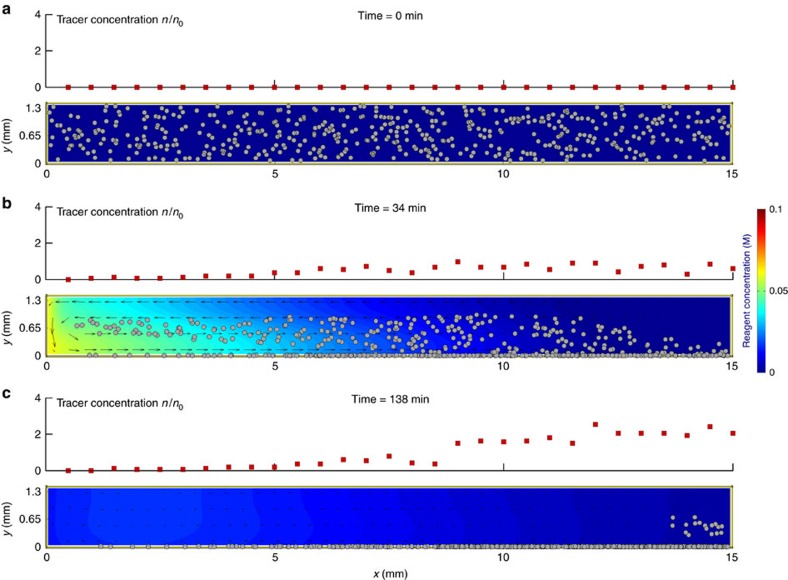
Stages of convective transport. (**a**) Initially, tracers are uniformly dispersed throughout the domain and the hydrogen peroxide concentration (shown by the colour bar) is zero. (**b**) The reagent, diffusing through the left wall, produces density variations resulting in a fluid flow that transports tracers. The catalyst at the bottom wall decomposes the reagent, creating a concentration (density) gradient. (**c**) After the reagent is consumed, the flow stops with areal concentration of tracers *n*/*n*_0_ increasing toward the right wall as shown by the red squares. (The red squares form a histogram, where the width of one bin corresponds to 500 μm.) Simulations were performed for *C*_0_=0.1 M and *r*_max_=1.7 × 10^−5^ mol m^−2^ s^−1^.

To better illustrate the physical mechanism, we first consider a situation where cargo is transported across the entire length of the microchannel. This happens when the reaction rate is relatively small, *r*_max_=1.7 × 10^−5^ mol m^−2^ s^−1^. The initial homogeneous distribution of spherical polystyrene tracers in the domain with zero reagent concentration is shown in Fig. 2a. Hydrogen peroxide entering the domain on the left increases the solution density relative to pure solvent and thus, due to gravity, generates downward convective flow, which spreads out horizontally along the bottom surface. The decomposition of reagent along the bottom plane results in the reagent concentration decreasing from the left to right wall, as indicated by the colour map in Fig. 2b. The corresponding density variations generate the fluid flow, which is marked by the arrows in Fig. 2b; this flow carries particles toward the right wall along the bottom plane. Due to the continuity of the flow, the less dense fluid at the right boundary rises upward and returns along the top plane back to the left boundary. The positions of propagating tracers are shown with grey markers in Fig. 2a–c for three successive time instances.

The tracer sedimentation velocity *V*=0.44 μm s^−1^, in the absence of convective flow, implies that, in the domain with height *H*=1.3 mm, all tracers should reach the bottom within 50 min. This value roughly sets the time during which tracers are carried by the flow. Sedimentation ensures that fluid flowing to the right in the lower half of the microchannel contains more tracers than the returning flow, which carries tracers to the left in the upper half of the channel. This asymmetry creates a net transport of tracers in the direction away from the entrance point of the reagent. Since we neglect friction between particles and domain walls, tracers that have already reached the bottom continue to move with the averaged local fluid velocity **u**(*x*, *R*, *t*), unlike in experiments.

The efficiency of the convective transportation can be characterized by the local areal concentration *n*=*m*/(Δ*x*Δ*z*), defined as a number of tracers, *m*, in a region of area Δ*x*Δ*z* in the bottom plane. Here Δ*z* represents a small thickness of the computational domain (in the direction perpendicular to *x* and *y*), where the system can be considered to be uniform in the *z* direction. (Recall that the simulations are performed in two dimensions, implying that the system is uniform in *z*.) Relative to the uniform distribution *n*_0_=*N*/(*L*Δ*z*), the areal concentration 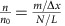 of tracers is calculated in bins of length Δ*x*=2.5 × 10^−4^ m along the bottom surface and plotted with red markers in Fig. 2a–c. (Note that *n*/*n*_0_ depends on the size of the bin Δ*x* and has a maximum value of *n*/*n*_0_=*L*/Δ*x*, describing the situation when all tracers are found in one bin of length Δ*x*.) Since tracers are only counted after they have sedimented to the bottom, the tracer concentration *n*/*n*_0_ generally increases with time. The increase is skewed, however, to the right side of the domain. This characteristic is due to convective transport of the tracers away from the source of dense reagent on the left. After the reagent has been consumed, the fluid flow stops and yields the tracer distribution shown in Fig. 2c. Note that tracers initially located to the right of the red mark are unaffected by the convective flow and simply sediment to the bottom. This leads to an average tracer areal concentration equal to the initial uniform value *n*_0_. Videos from the simulations depicting the dynamics of the tracer transportation are included in the Supplementary Information (Supplementary Movies 3–5).

### Parameters controlling transport properties

**Figure 3:**
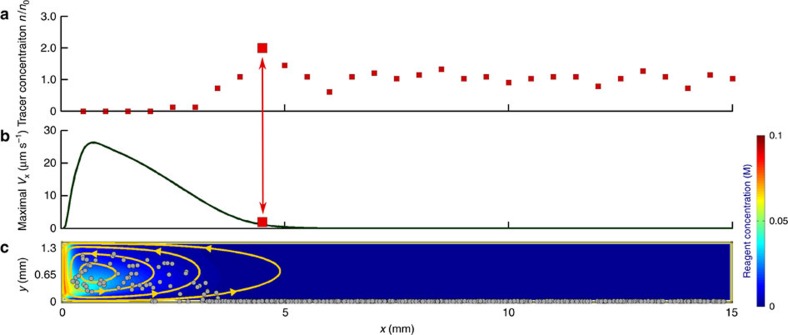
Formation of a maximum in the tracer distribution. (**a**) Areal tracer concentration *n*/*n*_0_, resulting from the fluid flow. (**b**) Maximal horizontal velocity goes to zero (red arrow) around the point where *n*/*n*_0_ is maximal (bold red square in **a**). (**c**) Convective vortex (yellow streamlines) dragging tracers along the bottom and aggregating them into the pile shown in **a**. The concentration of reagent, consumed by the catalyst along the bottom surface, is indicated by the colour bar. Simulations were performed for *C*_0_=0.1 M and *r*_max_=1.7 × 10^−2^ mol m^−2^ s^−1^.

When the reaction rate is relatively large (*r*_max_=1.7 × 10^−2^ mol m^−2^ s^−1^), the areal concentration of tracers *n*/*n*_0_ demonstrates a pronounced peak (large red square in Fig. 3a) that results from the structure of the convective vortex, which is marked in yellow in Fig. 3c. The fast depletion of reagent in the solution above the bottom surface limits the spatial extent of the density variations and the resulting convective flows. Thus, the suspended tracers are transported by the flow to the edge of the convective vortex, where the horizontal component of the velocity approaches zero (large red square in Fig. 3b). Here the tracers have aggregated into a pile. The correlation between the peak in *n*/*n*_0_ (red square in Fig. 3a) and zero maximal horizontal velocity (red square in Fig. 3b) is emphasized by the red arrow.

**Figure 4:**
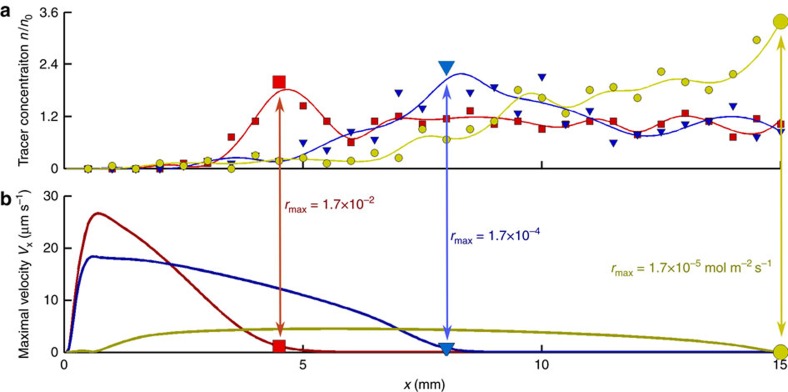
Reaction rate controls the position of maximum of tracer distribution. (**a**) Tracer areal concentration *n*/*n*_0_ as a function of the position along the channel. Decreasing *r*_max_ shifts the maximum values of *n*/*n*_0_ (bold markers) to the right. (**b**) The low reaction rates provide slower velocities, but enable fluid to flow throughout the entire domain (yellow line) and transport cargo from the left to the right wall. Vertical arrows and bold markers in **b** emphasize the correlation between the position of the maximum values of *n*/*n*_0_ (bold markers in **a**) and the distance at which horizontal flow speeds become low. The latter dots roughly indicate the edge of the convective vortex (shown in Fig. 3c) that drives the aggregation of the tracers into a pile.

When the reaction rate is decreased, the depletion of the reagent in the solution above the bottom surface becomes slower; this causes the reagent concentration and the generated flow to extend farther along the channel. The more elongated convective vortexes (with horizontal velocity shown in Fig. 4b) form piles of tracers farther from the left wall. Figure 4a depicts the shift of the maximum (large symbols) of the areal concentration *n*/*n*_0_ for a decreasing sequence of three representative reaction rates: *r*_max_=1.7 × 10^−2^, 1.7 × 10^−4^ and 1.7 × 10^−5^ mol m^−2^ s^−1^. To indicate the influence of the convective vortex on the spatial distribution of the tracers, we draw arrows between the position of maxima in *n*/*n*_0_ and corresponding positions in the plots of the in the horizontal velocity field. The maxima in *n*/*n*_0_ coincide with the edge of the respective convective vortex, at which the horizontal flow becomes negligible.

**Figure 5:**
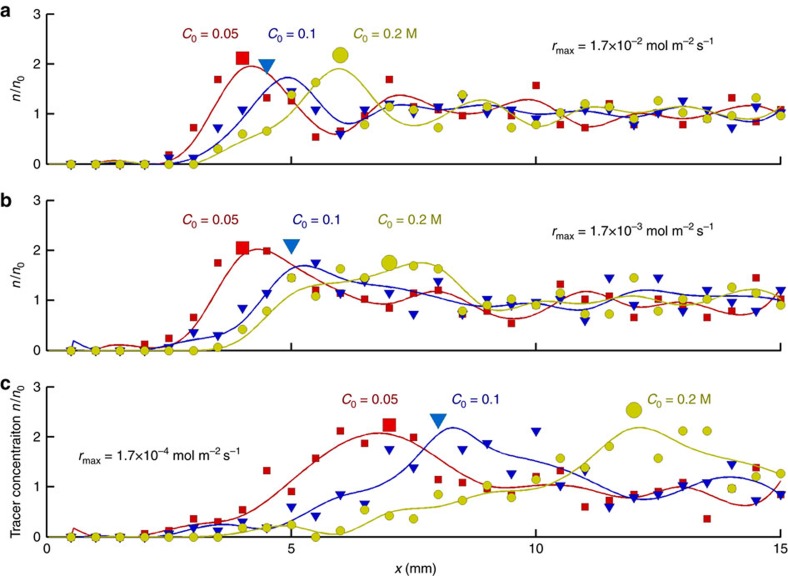
Amount of reagent controls position of maximum of tracer distribution. Increasing amount of reagent entails a longer time for complete decomposition during which the fluid flow reaches farther along the channel and aggregates tracers into piles (characterized by maximum of *n*/*n*_0_) farther away from the fuel entrance location at *x*=0. Shift of *n*/*n*_0_-maximum caused by increasing values *C*_0_=0.05, 0.1 and 0.2 M is demonstrated for the reaction rates: (**a**) *r*_max_=1.7 × 10^−2^, (**b**) 1.7 × 10^−3^ and (**c**) 1.7 × 10^−4^ mol m^−2^ s^−1^.

At constant catalyst coverage, the position of the maximum of the areal tracer concentration *n*/*n*_0_ can also be shifted by changing the amount of the reagent injected into the microchannel. Figure 5a–c shows the effect of changing the initial reagent concentration (from *C*_0_=0.05, *C*_0_=0.1 to *C*_0_=0.2 M) on the value of areal tracer concentrations *n*/*n*_0_ at three different reaction rates (*r*_max_=1.7 × 10^−2^, 1.7 × 10^−3^ to 1.7 × 10^−4^ mol m^−2^ s^−1^). Consumption of larger amounts of reagent takes longer and, therefore, the fluid flow reaches farther along the channel, shifting the maximum of *n*/*n*_0_ away from the fuel entrance at *x*=0.

Figure 3, 4, 5 clearly reveals that a high concentration of particles can be shifted from the entrance of the microchamber to a specified location in the device. (While the peak has a finite width, the particles have nonetheless been controllably displaced from the left edge to a specifiable region on the surface.) Hence, if a microscale sensor is placed at a particular location in the surface, the species can be carried and concentrated at that location. By delivering relatively high concentrations of analytes to the point of detection, the effectiveness of the device can be improved.

### Experimental results on convective pumping

To validate the above predictions on the directed transport of microparticles within the fluid-filled chambers, we performed experiments using two different catalytic micropumps. The first pump was based on the catalytic decomposition of hydrogen peroxide (H_2_O_2_) by platinum; the second involved the catalytic hydrolysis of urea by the enzyme urease. The very different pump systems (metal-based and enzymatic) were chosen to demonstrate the generality of our predictions that the transduction of chemical energy in these systems can lead to directional fluid pumping and targeted particle delivery.

**Figure 6:**
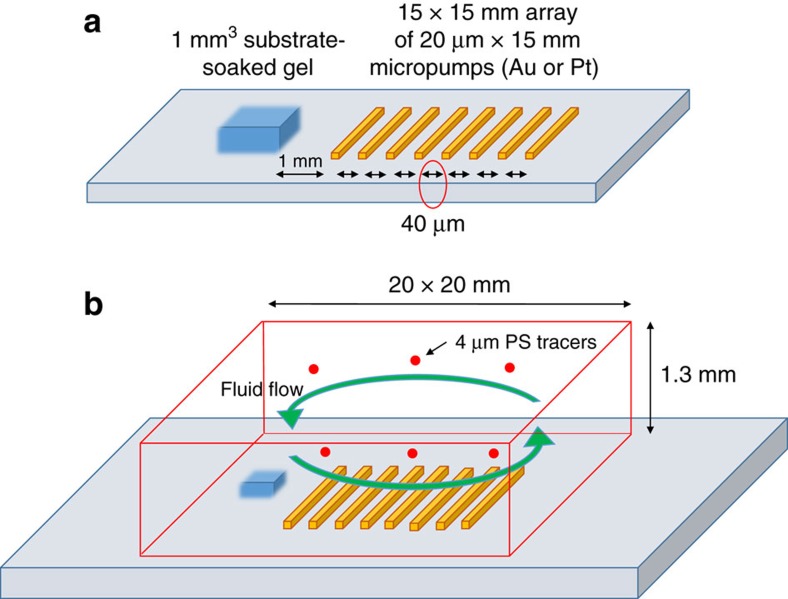
Schematic of the pump arrangement. (**a**) The micropump system consists of an array (15 mm × 15 mm) of ∼200 catalytic strips of width 20 μm that are arranged in parallel with each other with a distance of 40 μm between them on a glass slide. (The image is not drawn to scale.) An agar gel (1 mm^3^) is soaked in the desired reagent and placed perpendicular to the array at a distance of 1 mm. (**b**) To observe the fluid flow, 2 μm (radius) fluorescent polystyrene tracer particles in buffer or deionized water were introduced into the system and the system was sealed.

Individual pump strips, 20 μm in width and 15 mm in length with an inter-strip spacing of 40 μm, were patterned in a square array on a glass surface (see Fig. 6a for details). These strips were composed of 10 nm-thick platinum for the inorganic system. For the enzyme-based micropumps, urease was immobilized on 50 nm-thick gold strips, with the same array dimensions described above, using a biotin–streptavidin linkage (see Methods). A cylindrical spacer (20 mm in diameter and 1.3 mm in height) was placed on top of the pattern. (As noted above, such closely spaced stripes within a tall chamber should act much like a surface with a uniform coverage of catalyst.)

Sulfate-functionalized fluorescent polystyrene (sPsL) particles (2 μm radius) were used to visualize the fluid flow and quantify particle transport. Symmetry in the system was broken by placing an ∼1 mm^3^ reagent-soaked agar gel ∼1 mm away from the micropump pattern (Fig. 6b). Reagent diffuses into the surrounding fluid from the gel, resulting in a concentration gradient across the micropump pattern. The system was sealed and observed with a fluorescence microscope after ∼5 min of equilibration time.

#### Platinum pump

A gel soaked in H_2_O_2_ was used to set up a gradient of reagent at one end of the platinum array. As predicted from the simulations, the tracer particles were pumped away from the gel and the pumping velocity decreases with decreasing reagent concentration. Experimentally, the tracer particle speed decreases from 9.6±1.1 μm s^−1^ at 0.29 M H_2_O_2_ to 3.4±0.5 μm s^−1^ at 0.15 M H_2_O_2_. The particle speed was measured at a distance of 1 mm from the source of hydrogen peroxide, at a height of 50 μm above the surface (see Supplementary Note 2 for height calibration information). The motion of particles was observed at two different heights for each experiment to monitor both the forward flow along the glass surface and the return flow above the surface, as dictated by fluid continuity. The forward flow of tracer particles was observed at 50 μm above the surface, as mentioned above, for both the platinum and the urease pumps. The return flow (in the opposite direction to the forward flow) was observed at 500 μm above the Pt pattern (see Supplementary Movie 6). In the absence of H_2_O_2_, fluid flow was not observed for the Pt system (Supplementary Movie 7).

#### Urease pump

The urease pump showed similar behaviour to the platinum-based system. Namely, in the presence of urea, a reagent gradient perpendicular to the array gave rise to unidirectional fluid flow away from the gel. The tracer particles were pumped away from the gel near the surface of the slide, with a return flow further above the surface due to fluid continuity (Supplementary Movie 8). As predicted, the tracer particle speed shows reagent concentration dependence, decreasing from 6.5±0.4 μm s^−1^ at 500 mM urea to 2.8±1.1 μm s^−1^ at 250 mM urea. This tracer particle speed was measured at the beginning of the pattern, at a distance of 1 mm from the reagent source. In the absence of reagent, fluid flow was not observed (Supplementary Movie 9). These speeds were observed 50 μm above the pattern surface, comprising the forward flow of the convective loop. Similar to the platinum system above, flow in the opposite direction was observed at an increased height from the surface, consistent with fluid continuity.

### Spatial variations in pumping

**Figure 7:**
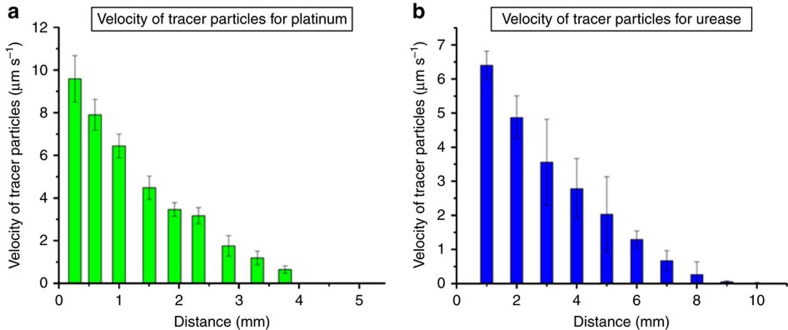
Spatial profile of the pumping speed of 2 μm sPsL particles. The plot in (**a**) is for the platinum system and the plot in (**b**) is for the urease system. The platinum system used an agar gel soaked in 0.29 M H_2_O_2_ to create a reagent gradient, while the urease system used a gel soaked in 500 mM urea. Error bars are calculated by taking the s.d. of the speed of 30 particles in a viewing window.

Variation in fluid-pumping speed was investigated across the surface. In agreement with the simulations (Fig. 3b), the experiments reveal that the tracer particles slow down as they move away from the gel, and, at a certain distance, the fluid flow stops and tracer particles show only Brownian diffusion. For pump arrays with 10 nm Pt deposited on the surface and a reagent gradient generated by a 0.29 M H_2_O_2_-soaked gel, the distance at which fluid flow dies off is 4 mm away from the gel. For gold patterns soaked in an enzyme concentration of 3 μM urease and a reagent gradient generated by a 500 mM urea-soaked gel, the distance is 10 mm away from the gel. This behaviour is a result of a combination of the differences in reaction rates between platinum and urease catalysis and differences in densities and diffusion rates of the chemical species in each system. The spatial profile of the tracer velocity for the two different systems is demonstrated in Fig. 7. The velocity profiles qualitatively agree with the spatial decay of the maximal horizontal velocities presented in Fig. 3b. As shown in the simulations, the distance at which the fluid flow stops and Brownian diffusion dominates depends on the reaction rate and resulting reagent depletion. In regions where the reagent concentration is small, the fluid density variations that generate convective flows are also small and the fluid velocity goes to zero.

### Convection-assisted delivery of microparticles

**Figure 8:**
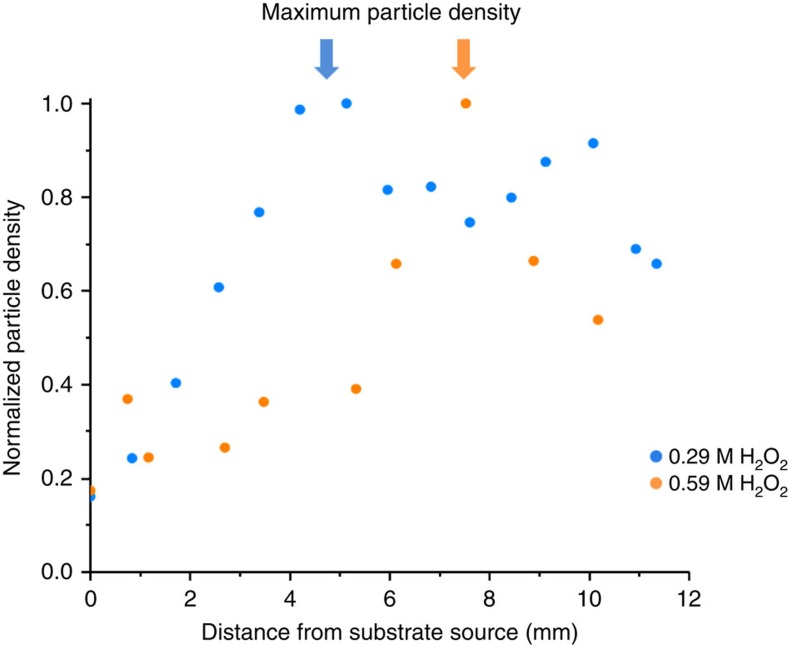
Density distribution of 2 μm sPsL particles for the platinum pump. The reagent concentrations are 0.59 M H_2_O_2_ (orange) and 0.29 M H_2_O_2_ (blue). For ∼0.5 mm^2^ areas along the centre of the pattern, the number of particles settled on the surface was determined by image analysis software as the per cent area covered by particles, which was then normalized. A fivefold increase in tracer particles at a distance of ∼8 mm from the reagent source was observed for 0.59 M H_2_O_2_. In contrast, for 0.29 M H_2_O_2_, the location of the maximum changed to ∼4 mm. The observed shift in maximum for the tracer density distribution agrees qualitatively with simulated shifts presented in Fig. 5.

**Figure 9:**
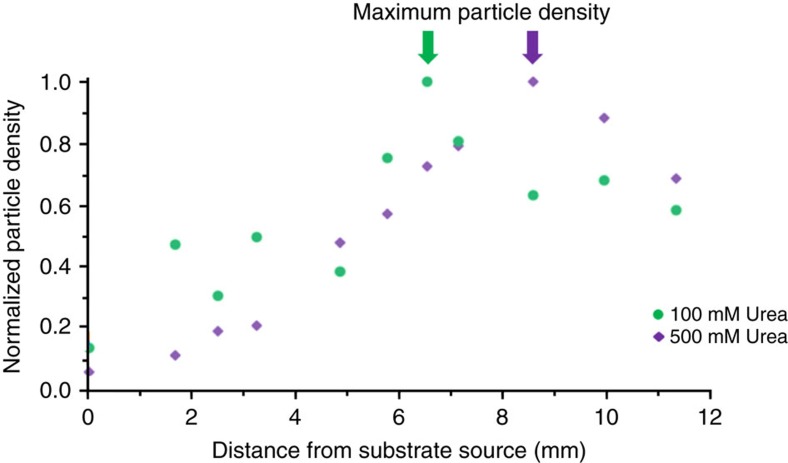
Density distribution of 2 μm sPsL particles for the urease pump. The data are for 100 mM urea (green) and 500 mM urea (purple). For ∼0.5 mm^2^ areas along the centre of the pattern, the number of particles settled on the surface was determined by image analysis software as the per cent area covered by particles, which was then normalized. For 100 mM urea concentration, a fivefold increase in tracer particles was observed at a distance of ∼6 mm away from the reagent source. Increasing the urea concentration to 500 mM changed the maximum to ∼9 mm away from the reagent source, with a 10-fold increase in particle density. The observed shift in maximum for the tracer density distribution qualitatively agrees with simulated shifts presented in Fig. 5 with increasing amount of reagent.

As indicated by the simulations, the decrease in tracer speeds across the surface and subsequent tracer sedimentation can be exploited for the directed delivery of microparticles. There exists a uniform distribution of tracers in the microchamber at the start of the experiment; however, at the end of an hour, all the tracers have settled onto the surface. Depending on the experimental conditions, sPsL tracers, which serve as analogues for general micron-sized analyte particles, can be focused at specific locations on the surface of the glass slide (Supplementary Movies 10 and 11). The platinum system displays a fivefold increase in the density of tracer particles (relative to the concentration at the source) at a distance of 4 mm away from the gel for 0.29 M H_2_O_2_ (Fig. 8). For the urease system, a 10-fold increase in the density of tracer particles was observed at a distance of 10 mm away from the gel for 500 mM urea (Fig. 9). The concentration of tracer particles was calculated using image analysis software (Supplementary Note 3).

Figures 8 and 9 also highlight our observation that the specific distance at which the microparticles are concentrated can be tailored by varying the conditions of the system. For the inorganic platinum-based array, the thickness of the catalyst deposited on the strip was held constant at 10 nm, while the reagent concentration in the gel was varied from 0.29 to 0.59 M H_2_O_2_ (Fig. 8). For the enzyme-based micropump array, the soaking concentration of urease for the array was kept constant at 3 μM, while the urea concentration in the gel was varied from 100 to 500 mM (Fig. 9). The simulation results in Fig. 5 indicate how an increase in the amount of reagent shifts the maximum for the tracer density distribution further away from the point of entrance, at all simulated reaction rates. Notably, a similar shift further away from the gel is seen in Figs 8 and 9, which show the experimental results when the reagent concentration was increased.

The particle density curve for 100 mM urea is not as smooth as for 500 mM urea. The reaction rate for urease increases until the urea concentration reaches ∼80 mM, beyond which it plateaus^28^,^29^. Indeed, the maximum urea concentration in the chamber never reaches the initial gel concentration of 100 mM due to incomplete diffusion from the gel. The variability in reaction rate at the lower urea concentration (likely ≤80 mM in the chamber due to slow diffusion) is likely the reason for the lack of smoothness in the particle density curve for 100 mM urea, relative to the 500 mM urea curve.

## Discussion

In summary, through a combination of modelling and experiments, we created a system where catalytic surface reactions produce unidirectional fluid flows that convey micron-sized particles to specific regions on the surface of microchambers. The introduction of reagents at one end of the chamber generated density variations within the fluid along the length of the chamber, producing asymmetric flow fields that are necessary for directed transport. The consumption of the reagent and resulting sedimentation of the dissolved particles led to the localized surface deposition. A powerful feature of the system is that the flow profile, and hence the location at which the particles are concentrated, can be tuned by altering the catalytic turnover rate and the concentration of the added reagent.

While we used a polymer gel as the reservoir of reagent, other materials (for example, porous membranes) could also be used for this purpose. Once all the reagents have been consumed, these reservoirs can be re-stocked with chemicals and, thus, the system can be used multiple times. In addition, the reservoir can be placed at different locations in the chamber, providing distinct ways to introduce spatially varying chemical gradients into the system. This flexibility in the design of the system opens new routes to fabricating complex microstructures or patterned surfaces. For instance, by tailoring the amount of reagent in sequential applications of the device, one can create complicated morphologies on the surfaces in a step-wise manner. Moreover, the tunable, active transport by catalytic pumps demonstrated here permits significant amounts of particulates to be delivered to sensors on surfaces and, thus, enables highly sensitive assays to be performed both efficiently and rapidly in micron-scale platforms.

## Methods

### Fabrication of micropump array patterns

The square arrays, consisting of ∼200 thin strips of metal spaced evenly, were fabricated over glass slides in the Nanofabrication Laboratory of Materials Research Institute at the Pennsylvania State University using standard lithography and deposition techniques. Glass slides are first cleaned with acetone and air-dried, then spin-coated with 5 ml of SPR-955 photoresist (Microposit) at 500 r.p.m. for 10 s and then at 3,500 r.p.m. for 60 s. This was followed by soft-baking the coated slides over a hot plate at 95 °C, for 1 min. The array geometry was modelled in CAD and printed over a chrome-on-glass mask (Nanofabrication Laboratory, Materials Research Institute, Penn State). For photolithography, the mask was placed in direct contact with the photoresist over the glass slide. The resist was then exposed to ultraviolet radiation for 12 s in a Karl Suss MA/BA6 Contact Aligner. The exposed wafers were post-baked for 1 min over a hot plate at 100 °C to cross-link the exposed film. MF CD26 developer was used to remove unexposed SPR-955 from the slide. The mould was developed for 2 min while being agitated, followed by washing it thoroughly with deionized water. After the glass slides were dried with a nitrogen blower, a 5 nm-thick layer of chromium and either a 50 nm layer of gold or a 10 nm layer of platinum was deposited on them using electron-beam evaporation using the Semicore Evaporator. Once the gold or platinum had been deposited, the glass slide was sonicated in a solution of dimethyl sulfoxide to remove the resist layer with gold or platinum on top. This left only the gold or platinum deposited on the glass slides in the desired pattern, shown in Fig. 6. For enzyme-based micropump arrays, enzyme was attached to the gold stripes, as fabricated above, using a biotin–streptavidin interaction.

### Enzyme immobilization on arrays

To achieve the biotinylation of enzymes, a urease solution (2 mg ml; urease from *Canavalia ensiformis,* Jack bean, Sigma Aldrich) was prepared with 100 mM PBS as the buffer. Before completing the total volume of the enzyme solution, a certain volume of a 0.1 mM biotin solution was added to get a ratio of 4:1 enzymes:biotin. The biotin linker used was 3-(*N*-maleimidylpropionyl)biocytin (Santa Cruz Biotechnology) for targeting the cysteine residues in urease. The mixture was reacted for 2 h at room temperature. Dialysis of the final solution was carried out at 4,500 r.p.m. for 5 min, followed by multiple washes with 10 mM PBS. The solution obtained at the end was diluted and stored at 4 °C until needed.

Another step involved the biotinylation of Au arrays for the enzyme immobilization. Biotinylation of the Au arrays was achieved by following a procedure reported previously^30^, (through the formation of a self-assembled monolayer using a biotin-thiol linker). For the preparation of the linker, a sulfhydryl-reactive biotinylating agent, Biotin HPDP (Apexbio), was reacted with a phosphine compound to form a thiol end group. Specifically, Biotin HPDP (1 mg per 2 array assemblies) was dissolved in dimethyl formamide (0.13 mg/ml) through sonication for 3 minutes at 45 °C, and tri-butylphosphine solution (Sigma) (5 μl per mg of biotin) was added. The reaction mixture was allowed to react for 30 mins at 45 °C, after which it was dissolved in a solvent mixture of 1:1 H_2_O:Ethanol (8 ml of solution per mg of biotin). This was the soaking solution for the Au arrays. The arrays were then incubated overnight at room temperature. After incubation, the arrays were washed several times with deionized water, followed by 10 mM PBS buffer. The self-assembled monolayer-modified surfaces were then incubated in a streptavidin solution (9 μM in 10 mM PBS) for 3 h, after which the surfaces were washed with 10 mM PBS buffer. Incubation of streptavidin-containing surfaces with enzyme-biotin solution was performed for 3–4 h before the experiments. The enzyme-functionalized surfaces were thoroughly washed with 10 mM PBS to remove any unbound enzyme molecules from the surface.

In the case of the urease pump experiments, secure-seal hybridization chambers were used to create a closed system on top of the urease-immobilized arrays. Solutions of 10 mM PBS buffer with sPsL particles (2 μm radius) (Polysciences Inc.) were used to visualize the fluid flow and quantify particle transport. Symmetry in the system was broken by placing a reagent-soaked agar gel near the micropump pattern (Fig. 6). Reagent diffuses into the surrounding fluid from the gel, resulting in a concentration gradient across the micropump pattern.

### Sample preparation

A 1% agar gel (Alfa Aesar) is prepared and then soaked overnight with reagent. For the platinum micropump arrays, the reagent is hydrogen peroxide (CCI). For the enzyme systems on the gold patterns, the reagent is urea (Sigma Aldrich). The reagent-soaked gel (∼1 mm × 1 mm × 1 mm) was placed perpendicularly to the micropump array pattern on the glass slide, at a distance of ∼1 mm. It should be noted that the gel varies in exact shape, size and location between experiments, which prevents the calculation of standard deviations between experiments. The whole system (gel and micropump array pattern) was covered with a secure-seal hybridization chamber (Electron Microscopy Sciences) with dimensions of 20 mm diameter and 1.3 mm height to establish a closed system. Care was taken to not damage the gel. To monitor the fluid flow, sPsL particles (2 μm radius) were introduced as tracers suspended in deionized water or PBS. The tracers were fluorescent with excitation of 580 nm and emission of 605 nm. To measure fluid-pumping velocity in each experiment, 30 tracer particles were tracked using the Tracker software (Cabrillo College).

Once the system was closed by the hybridization chamber seal and the tracer solution was introduced, observation of the system began after ∼5 min of equilibration. A microscope with optical and fluorescence capabilities was used to view the system, using a × 10 objective. Videos were recorded for a minute at ∼8 f.p.s., using an NIS-Elements camera, at different distances from the gel along the micropump pattern to determine the relationship between distance and pumping speed (tracer velocities). Videos were also recorded at different times over the course of ∼1 h at these various distances to measure the relationship between time and pumping speed. After ∼1 h, tracers begin to sediment to the surface of the glass slide, and a video was recorded to show the particle density distribution at the surface (see Supplementary Note 3 for more details). All experiments were conducted in triplicate.

### Data availability

The authors declare that all data supporting this work are contained in graphics displayed in the main text or in the Supplementary Information. Data used to generate these graphics are available from the authors upon request.

## Additional information

**How to cite this article:** Das, S. *et al*. Harnessing catalytic pumps for directional delivery of microparticles in microchambers. *Nat. Commun.* **8,** 14384 doi: 10.1038/ncomms14384 (2017).

**Publisher's note:** Springer Nature remains neutral with regard to jurisdictional claims in published maps and institutional affiliations.

## Supplementary Material

Supplementary Information Supplementary Notes and Supplementary Figures

Supplementary Movie 1 The video (a snapshot is shown in Supplementary Figure 1) shows convective transport of 500 tracers in a three-dimensional rectangular domain (3 mm × 1 mm × 3 mm). The enzyme (catalase) on the bottom plane coats a pattern consisting of parallel stripes oriented along the z-direction (similar to the experimental setup shown in Fig. 6). The stripe width and inter-stripe spacing are 50 μm, which differs only slightly from the experimental distances. A source of hydrogen peroxide, used as a fuel, is located on the left wall of the domain. The maximum reaction rate per unit area on the stripes is *r*_max_= 1.7×10^−3^ molm^−2^s^−1^.

Supplementary Movie 2 The video (a snapshot is shown in Supplementary Figure 2) shows convective transport of 500 tracers in a three-dimensional rectangular domain (3 mm × 1 mm × 3 mm) with catalase homogeneously coating the whole bottom plane. The reaction rate per unit area on the bottom is *r*_max_= 0.85×10^−3^ molm^−2^s^−1^, which is half of the rate used on the stripes in Supplementary Movie 1. Since the catalytic stripes in Supplementary Movie 1 only cover half of the surface area of the bottom plane, the total reaction rate is the same in the two cases.

Supplementary Movie 3 The video shows results presented in Fig. 2 for *C*_0_= 0.1 M and *r*_max_= 1.7×10^−5^ mol m^−2^s^−1^. The results presented in Fig. 3, 4, and 5 for *C*_0_= 0.1 M at other reaction rates demonstrate similar behavior.

Supplementary Movie 4 The video shows results presented in Fig. 5 for *C*_0_= 0.05 M and *r*_max_= 1.7×10^−2^ mol m^−2^s^−1^. The results presented in Fig. 5 for *C*_0_= 0.05 M at other reaction rates demonstrate similar behavior.

Supplementary Movie 5 The video shows results presented in Fig. 5 for *C*_0_= 0.2 M and *r*_max_= 1.7×10^−2^ mol m^−2^s^−1^. The results presented in Fig. 5 for *C*_0_= 0.2 M at other reaction rates demonstrate similar behavior.

Supplementary Movie 6 The platinum pump is comprised of strips of platinum and the gel is soaked with 0.59 M H2O2. 2 μm (radius) tracer particles demonstrate the direction of the flow after 5 min. As we change the focal plane and move vertically in the system, the reversal of the direction of tracer particles is seen, due to fluid continuity.

Supplementary Movie 7 The platinum pump is comprised of strips of platinum and the gel is soaked with DI water. 2 μm (radius) tracer particles demonstrate the absence of the flow after 5 min, with the tracers undergoing Brownian diffusion.

Supplementary Movie 8 The urease pump is comprised of strips of gold with enzymes attached to them and the gel is soaked with 500 mM urea in phosphate buffer. 2 μm (radius) tracer particles demonstrate the direction of the flow after 5 min. As we change the focal plane and move vertically in the system, the reversal of the direction of tracer particles is seen, due to fluid continuity.

Supplementary Movie 9 The urease pump is comprised of strips of gold with enzymes attached to them and the gel is soaked with phosphate buffer only. 2 μm (radius) tracer particles demonstrate the absence of the flow after 5 min, with the tracers undergoing Brownian diffusion.

Supplementary Movie 10 The platinum pump is comprised of strips of platinum and the gel is soaked with 0.29 M H2O2. The video was taken after 60 min. As we move along the pattern, the settled tracers can be seen and the changes in their density can be measured.

Supplementary Movie 11 The urease pump is comprised of strips of gold with enzymes attached to them and the gel is soaked with 500 mM urea in phosphate buffer. The video was taken after 60 min. As we move along the pattern, the settled tracers can be seen and the changes in their density can be measured.
