# Supplementary material for: Harnessing catalytic pumps for directional delivery of microparticles in microchambers
Source: Nat Commun. 2017 Feb 17;8:14384. doi: 10.1038/ncomms14384 (PMC5321755; doi:10.1038/ncomms14384)
Supplement: Supplementary Information — Supplementary Notes and Supplementary Figures [file ncomms14384-s1.pdf]

Supplementary Note 1: Control Simulations for Stripes vs. Solid Patch

The overall rate of reaction, which determines the fluid and tracer particle motion, is dependent on the amount of catalyst present on the patch. One approach for adjusting the amount of catalyst on the patch is to immobilize the enzyme or platinum catalyst onto stripes or other patterns. The average catalyst density is then controlled by adjusting the spacing between stripes, for example. If the inter-stripe distance is small enough, then we expect the stripes to effectively act as a single, uniform patch of catalyst. We used simulations to compare these two patch designs, using a three-dimensional rectangular domain (3 mm × 1 mm × 3 mm) with an altered simulation geometry from that used in further simulations. We found that using a pattern of catalytic stripes (**Supplementary Movie 1**) results in qualitatively the same fluid flow as coating the entire bottom surface of the chamber with catalyst keeping the same total catalytic activity (**Supplementary Movie 2**). Since the stripes are not an important aspect of the pump, we considered uniform coverage of catalyst over the bottom chamber surface in the simulations presented in the main text.

In addition, the simulations shown in **Supplementary Movie 1** and **2** illustrate the symmetry of the motion of the transported particles. Although the chamber geometry is not radially symmetric around the reagent source, motion in the *x*-*z* plane is predominantly along the radial direction. To analyze the system in more detail, we focus on the vertical (*x*-*y*) cross section through the center of the reagent source, $z = 1.5$ mm. By symmetry of the setup, there is no out-of-plane fluid flow in this cross-section. Hence, for simulations presented in the main text, we consider a two-dimensional model that represents a thin, vertical slice of the three-dimensional chamber. The physical mechanisms and qualitative behavior of the experimental system can be understood by examining the two-dimensional model.

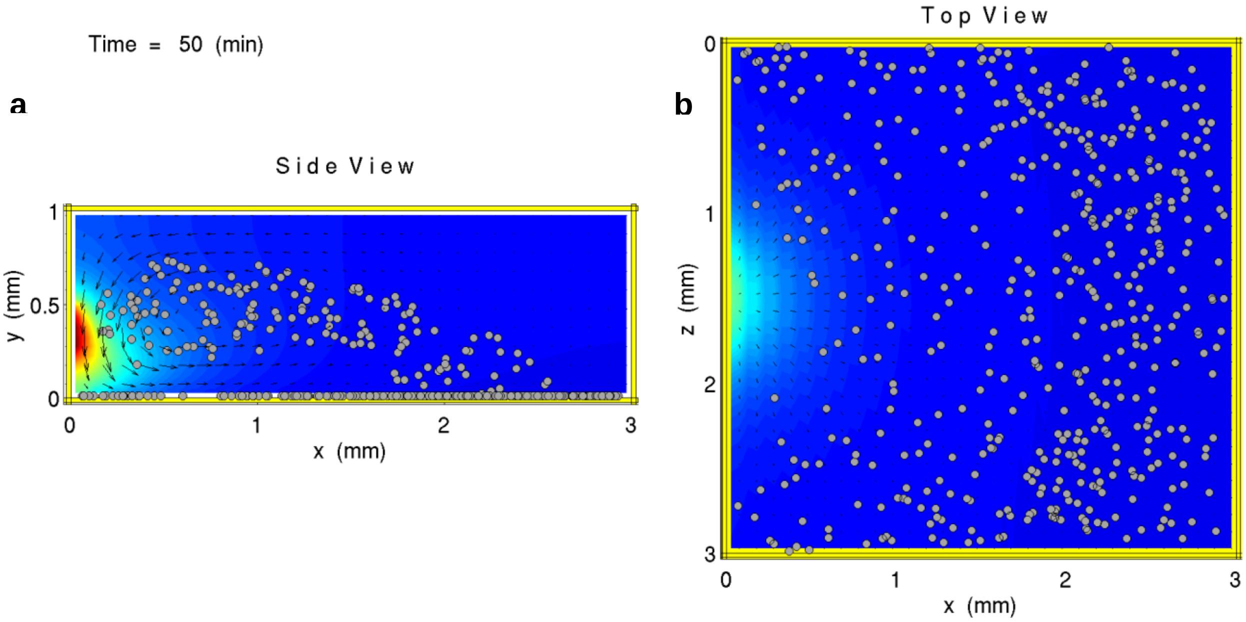

Supplementary Figure 1. The convective transport generated by enzyme coated stripes on the bottom. The enzyme (catalase) on the bottom coats stripes oriented along *z*-direction (similar to the experimental setup shown in Fig. 6). The stripe width and inter-stripe spacing are 50 µm. A source of hydrogen peroxide is located on the left wall of the domain. The maximum reaction

rate per unit area on the stripes is $r_{max} = 1.7\times10^{-3}$ mol m^{-2}s^{-1}. The left and right panels respectively show $z = 1.5$ mm and $y = 0.05$ mm cross sections of the simulation domain. The red and blue colors respectively indicate high and low concentrations of the reagent. Panels a and b show the respective side and top views of the simulation domain. The full time-dependent simulation is presented in the **Supplementary Movie 1**.

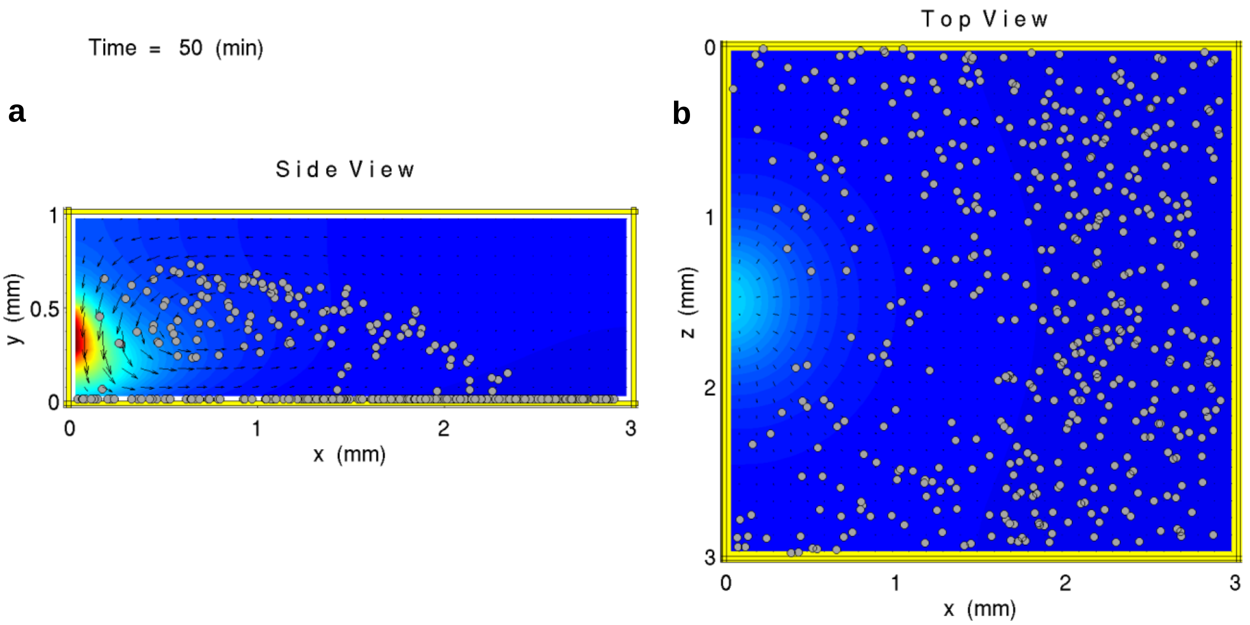

Supplementary Figure 2. The convective transport generated by enzyme uniformly coating the bottom plane. The enzyme (catalase) decomposing hydrogen peroxide coats the entire bottom plane where the maximum reaction rate per unit area is $r_{max} = 0.85\times10^{-3}$ mol m^{-2}s^{-1}. The left and right panels respectively show $z = 1.5$ mm and $y = 0.05$ mm sections of the simulation domain. The red and blue colors respectively indicate high and low concentrations of the reagent. Panels a and b show the respective side and top views of the simulation domain.

Supplementary Note 2: Microscope Details

Bright-field and fluorescence observation of tracers was made on a Nikon inverted microscope (Eclipse LV1000) fitted with an optical light source and CCD camera (Q-Imaging). The fluorescence mode of the Nikon Eclipse LV100 microscope was illuminated through green excitation band of a Nikon B2A filter cube. Videos were captured using an Andor Neo camera at a frame rate of 33 Hz. Nikon NIS Elements Imaging Software (V. 4) was used for video recording at a frame rate of ~8 fps.

The marks on the fine adjustment focusing knob of the microscope are correlated to heights above the surface by measuring the number of rotations of the adjustment knob required to transition from the surface of the chamber to the top of the chamber. When the bottom surface of the glass slide is in focus, the position on the focusing knob is recorded as 0 μm. When the top of the 1.3 mm deep chamber is in focus, the position on the focusing knob (including the number of

full rotations of the knob to reach this position) is calibrated as 1300 μm. This calibration allows for measurements of various heights using the focusing knob.

Supplementary Note 3: Density Distribution Calculation

The videos were analyzed using the image analysis software ImageJ (NIH) to determine the density distribution of tracer particles after they have settled on the inactive pump surface. The contrast threshold of the video is fixed in grayscale so that the tracer particles are shown as white pixels and the background is black pixels. The length scales are also calibrated to accurately represent the length scales on the glass slide. Finally, ImageJ is used to analyze the density distribution of the particles in a 0.8 mm^2 area at different points along the pattern; the percentage of area covered by the particles is determined by dividing the area comprised of white pixels (the tracer particles) by the area comprised of black pixels (the background surface). This percentage of area covered by tracer particles is then normalized in order to be able to compare density distributions between experiments, since tracer concentrations can differ from experiment to experiment.
